# Populist Attitudes in Adolescence are Measurable, Stable, and Linked to Political Trust: A Longitudinal Analysis of German High-School Students

**DOI:** 10.1007/s10964-026-02332-x

**Published:** 2026-03-06

**Authors:** Johanna Fee Ziemes, Katharina Eckstein

**Affiliations:** 1 University of Duisburg-Essen Universitätsstr, 2 | , 45141 Essen, Germany; 2 Friedrich Schiller University Jena, Humboldtstr. 27 |, 07743 Jena, Germany

**Keywords:** Political Socialization, Populist Attitudes, Political Trust, Longitudinal Analysis, Adolescence

## Abstract

Across the globe, populism has become a substantial challenge for democracies and educational settings alike. Populist attitudes include the belief that society can be sorted into two groups: The virtuous people and a corrupt elite. The study utilizes a sample of 1,206 German high-school students (52% female; *M*_Age, T1_=14.39; *SD*_Age, T1_=0.64) from two federal states (North Rhine-Westphalia & Thuringia). The stability of populist attitudes as well as their association with political trust over one school year are analyzed. Furthermore, connections to political efficacy, collective efficacy, and status anxiety in their role as potential precursors of populist attitudes are investigated. The results show that populist attitudes are cross-sectionally related to higher levels of status anxiety and internal political efficacy, but not to students’ collective efficacy. Longitudinal analyses further indicate that both populist attitudes and political trust remained generally stable over the course of a school year. Reciprocal effects linked higher levels of populist attitudes at T1 to lower political trust at T2 and vice versa, none of the other political attitudes (i.e., status anxiety, political efficacy, collective efficacy) predicted a change in either variable over time. Overall, the results indicate that populist attitudes are already conceptually distinct from (a lack of) political trust in mid-adolescence. The stability of these attitudes and their negative longitudinal links highlight the need to investigate populist attitudes early in life to better understand their development and to identify ways to reduce them.

**Supplementary Information:**

The online version contains supplementary material available at 10.1007/s10964-026-02332-x.

## Introduction

As populism has become a dominant mode of politics, understanding the formation of populist attitudes in young people as emerging citizens is an important task for everyone interested in political socialization. As their cognitive capabilities grow, young people learn that living in a society comes with specific rights and duties, and they form attitudes towards this social contract (Kornbluh et al., 2019). Populist parties and movements emphasize dissatisfaction with the current state of the social contract, undermine people’s trust in political institutions, and work towards a less pluralistic system, in which a wide variety of positions are no longer valid (Norris & Ingelheart, 2019; Sant, 2021). This article aims to foster a better understanding of the development of populist attitudes in emerging citizens by contrasting them with the related construct of political trust. Both populist attitudes and political trust are expressions of the relationship young people have with the political structure but carry different implications for both the individual development and the stability of pluralistic democratic systems. To promote a holistic understanding of the formation of populist attitudes and political trust, this study further considers adolescents’ views of their own position within the political system (i.e., political efficacy, collective efficacy) and their future-related concerns. These indicators were chosen because previous research identified feelings of helplessness as contributing factors to the emergence of populist movements (Sant, 2021). The present study investigates whether, and to what extent, these indicators serve as precursors of populist attitudes in adolescence as a formative state of political socialization. Using a German sample of adolescents, this study investigated the longitudinal interrelations of populist attitudes with political trust along with other potential precursors of populism, i.e. status anxiety, political efficacy, and collective efficacy.

### The Relevance of Populism in Youth for Societies

Populist attitudes in adolescence can have lasting consequences for political development (Eckstein, 2019). While people can and do change their attitudes and values at any age, those acquired in childhood and adolescence are particularly persistent and strongly influence attitudes in adulthood. Moreover, political events have been shown to affect young people strongly in their political development (Conzo & Salustri, 2019; Dinas, 2013). While emerging citizens grow up, their attitudes towards the government and its institutions evolve and become more concrete (Hess & Torney, 1967). Additionally, young people gain direct, first-hand experience with public institutions. This is reflected in theories of political socialization that emphasize everyday experiences within groups and institutions (i.e., family or school; Flanagan, 2013; Ziemes, 2025). While children and adolescents grow up, they internalize the rules of society and develop a sense of their own position within the system based on their daily experiences of participation, responsibility, or (lack of) inclusion. Populism has the potential to undermine social trust and, thereby, social cohesion by shaping young people’s perceptions of who belongs to the community and who does not (Filsinger, 2024). Therefore, understanding the early formation and stability of populist attitudes provides insights into the broader process of political socialization (Noack & Eckstein, 2023).

Populism is generally conceptualized as a thin-centered ideology. Thin-centered is a descriptive term that denotes that populism is compatible with different other political approaches, such as right-wing, left-wing, ecological, or religious movements (Mudde & Kaltwasser, 2015). No single measurement approach of populist attitudes has emerged (Castanho Silva et al., 2017), but three dimensions of populism are often distinguished: anti-elitism, a belief in the virtuousness of “the people”, and the support of unrestricted popular sovereignty (Schulz et al., 2018). Current research findings indicate that anti-elitism represents the primary dimension when capturing populist attitudes as a multidimensional construct among youth (Grunwald et al., under review). Anti-elitism generally describes an attitude that clearly delineates a construction of “the people” from “the elite”, simplifying social realities in the process by breaking down complex webs of belonging into those two categories.

Populism generally goes along with an anti-pluralist thrust, excluding everyone not considered to belong to a certain group, e.g., societal minorities (Mudde, 2004). Therefore, populism is strongly related to the process of identity formation, specifically to the construction of less complex identities. Social identity theory suggests that people can feel a sense of belonging to different social groups at the same time and that identity complexity is related to more outgroup tolerance while the strong identification with one group is related to more prejudice (Beelmann, 2020; Brewer & Pierce, 2005). Patterns of identity change how people engage with different groups and how information is processed. Populism is connected to a more intuitive mode of information processing, a stronger tendency towards a conspiracist mentality, and the acceptance of bullshit information (van Prooijen et al., 2022). Bullshit receptivity is a technical term that describes the tendency to find meaning in nonsensical statements. The construction of one’s own identity in opposition to an outside antagonist and the simplifying approach to knowledge seem to reinforce one another, with the mode of information processing bolstering the in-group cohesion and the group identity, guiding the assessment of information (Kinnvall & Svensson, 2022; Sant & Brown, 2021). In consequence, populism in this article is defined as an attitude (at the individual level) and a strategy (at the societal level) that fosters polarization by dividing the society into two groups: The ‘pure people’ and the ‘corrupt elite’ (Mudde, 2021; Sant, 2021). Populism is not only a challenge for education and the political socialization of students, but also for democracies. As populistic reasoning favors dichotomous constructions of identities and simplifies solutions, it counteracts the strength of democratic decision-making processes (Abs, 2021).

There is already some support for the assumption that populism can be measured in younger generations. One study using a large sample of teenagers between the ages of 12 and 18 from Austria, Germany, and Switzerland found that boys and students who experienced negative relationships with teachers were more likely to report populist attitudes (Jungkunz & Weiss, 2024). A different analysis from North Macedonia showed that populism, conspiracist thinking, and political radicalism were substantially correlated at the age of 19 (Kenig & Spasovski, 2023). Similarly, a study using a German sample of adolescents and young adults identified (expected) associations with related constructs, such as intolerance, conspiracy beliefs, political trust, and voting preferences (Grunwald et al., under review). In one of the few longitudinal studies conducted so far, moderate to high levels of stability were found over one year for populism’s subdimensions of anti-elitism, anti-pluralism, and sovereignty of the people among 14-year-olds (Körner et al., 2023). Overall, however, research on populism in youth remains limited, particularly regarding longitudinal studies examining its dynamics and influencing factors over time.

Populism already influences students’ educational processes. As discussed, students use the experiences made on the micro level (i.e., the school) to understand society as a whole. Specifically, experiencing devaluation and exclusion based on populist strategies can decrease students’ sense of belonging in school (Wray-Lake et al., 2018). Increased discrimination can be explained by a lessened awareness of societal inequality that can be found in people supporting populist actors (Dunn et al., 2022). Experiences of discrimination is generally connected to a decrease in academic performance and mental health (Civitillo et al., 2023). The populist mindset, its connection of identity, information processing, and uncritical opposition to outgroups, can be seen as a challenge to educational approaches that aim to foster intergroup tolerance and critical citizenship. Critical citizenship encompasses the ability to thoroughly assess the trustworthiness of institutions and potentially contribute to democratic reforms (Norris, 2011; Ziemes et al., 2019).

### Determinants and Correlates of Populist Attitudes

Populist attitudes are on the rise in many countries around the globe (Norris & Inglehart, 2019), and researchers have different estimations concerning the severity of the current increase. Some argue that the cultural backlash is primarily a problem of older generations and is likely to decrease over time (e.g., Norris & Inglehart, 2019), while others are not convinced that populism will disappear over time. Accordingly, although older generations are found to support authoritarian values more than younger ones, all generations are equally likely to hold populist attitudes (Schäfer, 2022). Moreover, Schäfer (2022) stresses the importance of distinguishing between populism and conceptually closely related political orientations, such as (lack of) trust in political institutions, to understand the political culture of a country.

#### Political trust 

Political trust is related to the critical evaluation of political institutions and connected to early psychosocial development. Erikson (1959/1994) argues that young children develop a foundational trust in the world and in the safety of their surroundings, while adolescence is the time in which they create meaning in their relationships with peers, other people, and the world around them. Trust in political institutions continues to develop throughout life, but it is strongly shaped by experiences in youth (Ziemes, 2025), such as political discussions and experiences at home or in school (Leggett-James et al., 2023; Tzankova et al., 2021). Political trust can be understood as an unwritten social contract through which people give others (e.g., political representatives) the authority to act on their behalf, expecting them to carry out their duties competently and honestly (Flanagan, 2013; Norris, 2022). Although emerging citizens do not consciously agree to this social contract, it is an important aspect of their relationship with all institutions and, indirectly, with older generations that hold institutional power. However, political trust should not be mistaken for unquestioning support. If the revocation of trust is based on an understanding of democratic processes and principles, this revocation can be a democratizing force that motivates people to work towards stronger and more accountable democratic institutions (Norris, 2011). Research investigating how young people bestow their trust demonstrates that emerging citizens can already take on the role of critical citizens by acquiring the necessary political competencies (Lauglo, 2013; Stals & Ziemes, 2024). The revocation of political trust may also foster solidarity with minorities and drive institutional change (Pappas et al., 2009; Rogers et al., 2024), countering right-wing populism, which tends to undermine support of minority rights (Huber & Schimpf, 2017). While people with strong populist attitudes tend to distrust all institutions they perceive as elite and not aligned with their agenda^1^, not all people who distrust institutions are populists. The relationship of trust and populism depends on the general relationship of each person with the political system and the perception of one’s relative position in it.

#### Political efficacy 

Populist attitudes are often connected to feelings of political insignificance on the part of the citizens. While strong political trust can be especially important when many people have no interest in political participation, a feeling of helplessness has been discussed to be a potent driver of populist attitudes (Şimşek, 2024, p. 666). Yet, to clearly position populist attitudes as a facet within a political mindset, it is important to distinguish between political efficacy and responsiveness. The former is generally referred to as internal political efficacy and describes one’s perceived ability to participate in decision-making processes. The latter, in turn, is termed external political efficacy, which describes the belief that the political system is responsive to such participation (Niemi et al., 1991). The current study will focus on the internal aspect of political efficacy.

In youth, internal political efficacy is a central facet of the political mindset and strongly related to the willingness to engage in civic activities (Diemer & Rapa, 2016; Eckstein et al., 2013) and to vote in elections (Maurissen, 2020). Moreover, internal political efficacy has been shown to be associated with higher levels of political trust and satisfaction with democracy (Bäck & Kestilä, 2009; Parent at al., 2005). While internal political self-efficacy is, therefore, generally seen as an attribute that strengthens students’ pro-democratic resilience, it may also be linked to stronger populist attitudes. Within populist rhetoric, there is a clear emphasis on narratives that highlight the capabilities of “the people”, asserting their individual and collective competence in contrast to that of “the elites” (Rico et al., 2020). A cohort study of Colombian youth demonstrated that internal political self-efficacy increased along with the acceptance of corruption, indicating that political efficacy is not inherently linked to pro-democratic values (Velez & Knowles, 2022).^2^

#### Collective efficacy 

While internal political efficacy relates to each individual’s capacity, collective efficacy is the conviction that people can drive change in political systems by working together (Yeich & Levine, 1994). This construct provides an important perspective, as even effective and competent individuals are unlikely to be able to change a system profoundly on their own. A significant part of the population working together, on the other hand, can achieve just that. Therefore, collective efficacy can be seen as a bridge between internal political efficacy and responsiveness (external political efficacy), as it relates to activities in which people can participate and to the belief that the system will appropriately respond to collective demands (Abrams et al., 2020). If people perceive strong social relationships with others, individual efficacy can translate into collective efficacy (Eidhof & Ruyter, 2022). Social cohesion includes intra- and intergroup connections (Chan et al., 2006). Intergroup connections weaken when societal inequality increases, and - as described above - populism makes it harder for different groups to reconcile and cooperate, worsening the very problem it claims to address (Jay et al., 2019; Oxendine, 2019). Despite its apparent relevance, empirical studies on the relationship between collective efficacy and populist attitudes are rare. Although anger, fear, and a strong ideological stance have been shown to increase people’s intention for collective action (Marinthe et al., 2025), these emotions are not equivalent to populist attitudes. Longitudinal analyses could shed light on the relationship between collective efficacy, political trust, and populism and show if the perceived ability to jointly influence politics may prevent political polarization.

#### Status anxiety 

Apart from internal and collective efficacy, status anxieties are discussed as another important driver of populist attitudes. The idea of status anxiety stems from the idea that all people estimate their own relative social status within society. For the development of populist attitudes, the absolute perceived social status is not as important as the expected trajectory; people try to avoid status loss and may support more radical parties if they feel their status is threatened (Gidron & Hall, 2017). The perceived threat to one’s own status can arise from economic crises but also from cultural shifts in social hierarchies, such as the de-stigmatization of racialized people, queer people, and women. Indeed, researchers have speculated that the “silent revolution” (Norris & Inglehart, 2019, p. 32) might be a driver of populist attitudes in the global north. The silent revolution refers to the fact that many minoritized groups have fought for and gained civil rights (e.g., interracial marriages, same-sex marriages) despite a significant part of the population disagreeing with these developments. Since populism builds on the belief that only one societal group truly represents the will of the true people (Müller, 2015), the growing recognition of societal pluralism can foster feelings of division and status threats. Even if marginalized groups do not fully reach the level of societal acceptance enjoyed by privileged groups, any reduction in status difference may be perceived as a threat to one’s position within the hierarchy. This effect can be exacerbated by economic crises and increasing societal inequalities (Layte & Whelan, 2014).

Young people are already aware of their societal status. Their expectations about the future - as well as perceived threats to it - shape their political attitudes (Deimel & Abs, 2024). In a cross-sectional study, future-related anxieties emerged as the strongest predictor of populist attitudes in a representative sample of Germans aged 14 and older, even after controlling for education and subjective economic deprivation (Roleder, 2024). A comparison of the International Civic and Citizenship Education Study (ICCS) from 2016 to 2022 shows that youth have become more likely to recognize global financial crises and unemployment as threats for the future (Schulz et al., 2023). Growing concerns may be one potential cause for the increasing susceptibility to populist narratives among emerging citizens, as may be worries about changes in traditional societal roles. In Europe, for instance, one in five students agreed that, in times of unemployment, men should have the right to a workplace over women (Abs et al., 2024), indicating a mindset of gender competitiveness.

#### Socio-demographic factors 

Right- and left-wing populist movements tend to be linked to the support of traditional gender roles (Dingler & Lefkofridi, 2021), which can be unappealing for girls and women who have fewer liberties in less egalitarian systems. In fact, one study found boys to exhibit markedly stronger populist attitudes (Jungkunz & Weiss, 2024). This gender difference might depend on the measure, sample, and context. Another study found girls to be slightly more prone to anti-elitism (Körner et al., 2023).

#### Apart from individual factors 

Regional characteristics and their opportunity structures should also be considered in the formation of populist attitudes. For example, populist attitudes have been associated to lower levels of educational attainment (Heiss & Matthes, 2017) and - in the case of Germany– were found to be more pronounced in regions of the former German Democratic Republic (GDR; Körner et al., 2023; Shell & Holding, 2019). Despite more than 30 years having passed since German reunification, it remains important to consider such regional differences, as former East and West Germany differ not only in their recent history but also in structural characteristics, such as levels of urbanization and ethnic-cultural diversity. Accordingly, observed differences in populist attitudes are discussed in terms of historical, structural, and individual factors (Weisskircher, 2020). Overall, the presented studies suggest that youth are susceptible to populist attitudes due to their limited rights and opportunities to shape an increasingly uncertain future.

## Current Study

**Fig. 1:**
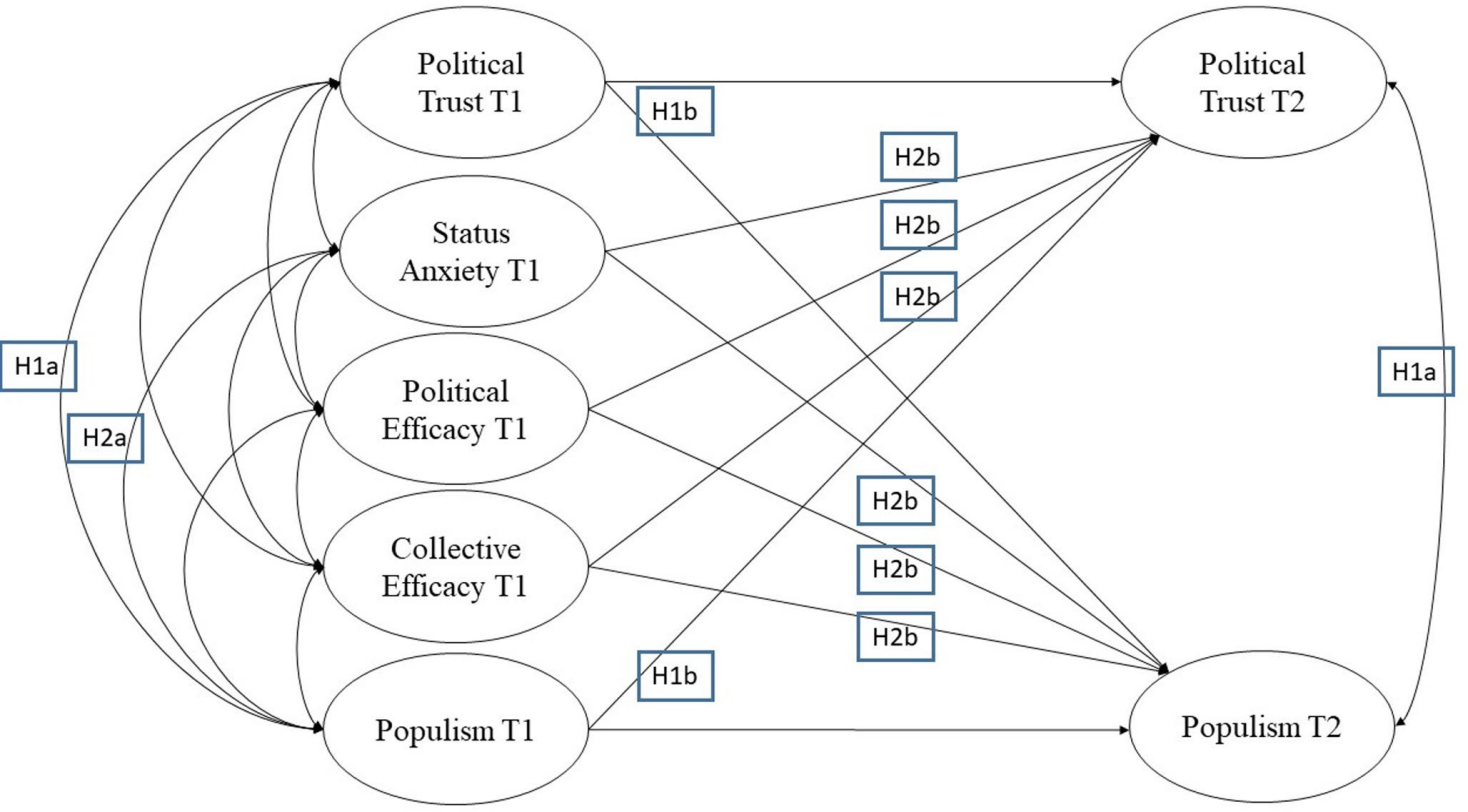
Illustration of hypotheses and intended analyses. Note. Gender, socioeconomic status, and federal state are introduced as predictors for all T1 variables and notshown to improve readability

While some studies already show that populist attitudes can be measured in adolescence, little is known about their development. With longitudinal analyses the directionality of effects can be asserted with some confidence. In addition, longitudinal analyses can offer insights about the stability of phenomena and security concerning the direction of effects. Accounting for the stability of populist attitudes can help to determine whether they are already deeply rooted in emerging citizens’ political mindsets and how resistant they are to change. While political trust combines social experiences with critical appraisal of institutions, populist attitudes combine dichotomous constructions of identity with unfounded skepticism against elites. For this study the relationship between populist attitudes and political trust will be investigated under the assumption that populist attitudes and political trust are substantially negatively correlated at T1, yet still represent separate constructs (1a), and that both affect each other across time (1b). These hypotheses are illustrated in Fig. 1. Furthermore, this study explores the role of populist attitudes within the political mindset. Specifically, it examines the roles of internal political efficacy, collective political efficacy, and status anxiety. As outlined above, research has shown inconclusive findings for the connection between efficacy beliefs and populist attitudes, whereas the role of status anxieties is comparably well established. Based on previous studies, considerable cross-sectional associations between status anxiety and populist attitudes are assumed (2a). Additionally, it is expected that status anxiety and a lack of efficacy beliefs at T1 predict higher populist attitudes and lower political trust at T2 (2b). Further connections are explored as shown in Fig. 1.

## Methods

### Sample

The data for this study was collected as part of a larger project aimed at assessing civic attitudes and behaviors in youth. The sample consisted of 1,206 German adolescents in Grade 9 (*M*_AgeT1_ = 14.39, *SD* = 0.64, range: 13–17 years) who were surveyed twice, at the beginning (T1, fall 2021) and end (T2, summer 2022) of the school year. The surveys were conducted in two regions of Germany: North Rhine-Westphalia (NRW; *n* = 489, 40.5%) and Thuringia (*n* = 717, 59.5%). The sample thus reflects contextual variations in terms of urbanization and cultural diversity. Unlike NRW, Thuringia was a part of the former German Democratic Republic (GDR), a region that is economically more deprived than the rest of Germany and in which Germany’s far-right populist party Alternative für Deutschland (AfD) receives stronger support than in the other federal states (Weisskircher, 2020). The participating students were recruited through their respective schools (i.e., emails, telephone calls, or class visits) and were enrolled in college-bound high schools (58.3%), practically oriented schools (16.3%), and in comprehensive schools (25.4%). Gender was roughly equally distributed (*n*_female_ = 624, 51.7%; *n*_male_ = 565, 46.8%; *n*_diverse_ = 9, 0.8%). Most students were born in Germany (*n* = 839, 69.6%), with a quarter of the sample being of immigrant descent (i.e., either students themselves or at least one parent were not born in Germany; *n* = 281, 23.3%; *n* = 86, 7.1% provided no answer).

Altogether, 31 schools and 89 classes were included in the study, and the number of students per class varied between 5 and 26 (*M* = 17.69, *SD* = 5.79). The procedure of data collection was similar for all schools: Once schools had agreed to participate, students and parents were asked for their consent. The survey was conducted during regular school hours. Students completed a questionnaire on different civic topics, which took approximately 90 min. During this time, a research assistant from the project was present in class to administer the survey. All classes received a small contribution to their class fund.

### Measures

If not otherwise indicated, the response options ranged from 1 = *I do not agree at all* to 5 *= I fully agree*. A complete summary of the scales’ item wordings is provided in the online supplemental material (Appendix A).

#### Populist attitudes and political trust 

Populist attitudes were assessed with five items (e.g., “The members of parliament lose touch with the people quite quickly.”; “The differences between the people and the so-called elite are much greater than the differences within the group of the people.” (adapted from Schulz et al., 2018). This scale has a stronger conceptual focus on the subdimension of anti-elitism than multidimensional instruments (Castanho Silva et al., 2017). Reliability analysis revealed that the internal consistency remained below the desired threshold at T1 (ω = 0.62), while it was acceptable at T2 (ω = 0.71). Political trust was assessed by three indicators (i.e., “How much do you trust … the government/ political parties/ the European Union”; adapted from Schulz et al., 2016). Response options ranged from 1 = *not at all* to 5 = *completely*. Mc Donald’s omega revealed good internal consistencies (ω_T1_ = 0.71, ω_T2_ = 0.70).

#### Political efficacy, collective efficacy, and status anxiety

Political efficacy was captured at T1 with three indicators **(**e.g., “I find it easy to take part in discussions about political topics.”; Noack & Marcek, 2018) and revealed a good internal consistency (ω = 0.77). Collective efficacy was measured at T1 with two questions (e.g., “I believe that young people can make things better by working together.”; *r* = .32; Noack & Macek, 2018). Finally, status anxiety at T1 was assessed with three questions (i.e., “When you think about your future, how much do the following things personally worry you?” – A parent losing a job or a deterioration in the family’s financial situation / not finding a training position / being financially worse off than one’s parents in the future). The response options ranged from 1 = *does not worry me at all* to 5 = *worries me a lot* (ω = 0.75).

#### Gender, region, and socio-economic status 

Gender (0 = *female*, 1 = *male*), region (0 = *NRW*, 1 = *Thuringia*), and family financial resources (Does your family have enough money for everything your family needs?; 1 = *not at all*, 4 = *completely*) were included as control variables in all main analyses. Since all students were in the same grade (9th grade), age was not treated as an additional covariate.

### Analytical Procedure

SPSS and R (Hallquist & Wiley, 2018; IBM, 2021; R Core Team, 2023) were used for the data preparation and M*plus* 8.7 for the main data analysis (Muthén & Muthén, 1998 –2018). The intraclass correlations of the study variables ranged between 0.024 and 057, indicating that only a limited share of variances was located at the classroom level^3^. This means that individual level analyses are most appropriate. Before proceeding to the main analyses, measurement invariance was tested in constructs assessed at two time points (i.e., populist attitudes, political trust) using confirmatory factor analysis (CFA) to compare configural, metric, and scalar models. To conduct our main analyses, structural equation modeling (SEM) was applied, which allowed investigating variable relationships at a latent level. Because of the clustered nature of the sample (i.e., students being nested within classrooms), M*plus*’s type=complex option was used, which corrects standard errors and chi-square statistics for non-independence of observations. Model fit was evaluated based on the χ^2^-statistic and standard values of common goodness-of-fit indices (Goodness of Fit Index (GFI) ≥ 0.95, Root Mean Square Error of Approximation (RMSEA) ≤ 0.06, Standardized Root Mean Square Residual (SRMR) ≤ 0.08; Hu & Bentler, 1999).

## Results

### Preliminary Analyses

As in most longitudinal studies, not all students participated in the survey at both time points (complete data: *n* = 1.017, 84.3% of initial sample). Little’s MCAR test (Little, 1988), which included all study variables, was significant (*χ*²[186] = 230.93, *p* = .014), suggesting that data were not missing completely at random. To gain a deeper understanding, follow-up analyses were carried out in which students with no missing data were compared to students who did not participate at T2. The results showed that missingness was related to political trust (*r* = .81, *p* = .005) and family financial resources (*r* = .86, *p* = .006), indicating that students with lower levels of political trust at T1 and lower family financial resources were more likely to drop out of the study than their peers. To prevent a further reduction of the initial sample size, missingness was addressed using a full information maximum likelihood approach (maximum likelihood estimation with robust standard errors, MLR; Jeličič et al., 2009).

Analyses of measurement invariance across time supported the existence of full metric invariance for both populist attitudes and political trust (i.e., equal factor loadings; populism: Δχ^2^ (4) = 3.17, *p* = .530, ΔCFI < 0.001, ΔRMSEA = 0.002; trust: Δχ^2^ (2) = 1.48, *p* = .477, ΔCFI < 0.001, ΔRMSEA < 0.001) and partial scalar invariance for populist attitudes^4^ (i.e., equal intercepts; Δχ^2^ (3) = 5.31, *p* = .150, ΔCFI = 0.001, ΔRMSEA = 0.001. For political trust, however, scalar invariance could not be confirmed; Δχ^2^ (1) = 12.54, *p* < .001, ΔCFI = 0.003, ΔRMSEA = 0.028. The items can, thus, be used to create a latent scale that reliably estimates correlation and regression coefficients within and between waves (Rutkowski & Svetina, 2014). The respective invariance constraints were, therefore, included in all subsequent analyses.

The zero-order correlations of the study variables are presented in Table 1. The findings indicate a substantial stability for both populism and political trust over time. As expected, status anxiety was positively associated with populist attitudes and negatively with political trust. Political efficacy and collective efficacy correlated positively with political trust but showed no association with populist attitudes. Students in Thuringia were more likely to support populist attitudes than students from NRW, whereas gender and socio-economic status were only weakly related to populist attitudes and political trust. Female students reported lower status anxieties and collective efficacy than male students. Overall, the results support the idea that political trust and populist attitudes fulfill different roles within the political mindset and that, even in adolescence, it is not perceived status per se but the fear of losing one’s status that is linked to populist attitudes.

**Table 1:** Zero-order correlations of study variables

	1	2	3	4	5	6	7	8	9
1. Populist attitudes T1	1								
2. Populist attitudes T2	0.652***	1							
3. Political trust T1	− 0.358***	− 0.344***	1						
4. Political trust T2	− 0.422***	− 0.459***	0.652***	1					
5. Status anxiety	0.204***	0.157***	− 0.174***	− 0.164***	1				
6. Political efficacy	0.128**	0.024	0.302**	0.189***	− 0.085*	1			
7. Collective efficacy	0.055	− 0.012	0.330***	0.245***	0.033	0.344***	1		
8. Federal state (1 = Thuringia)	0.208***	0.138**	− 0.096*	− 0.090*	0.098*	− 0.093	− 0.144**	1	
9. Gender (1 = male)	− 0.062	− 0.061*	0.088*	0.081*	− 0.201***	0.028	− 0.157***	− 0.027	1
10. Socio-economic status	− 0.092*	− 0.043	0.105***	0.091*	− 0.182***	0.066	0.069	− 0.045	− 0.044

Note. *N* = 1.206; type = complex. Scales are introduced as latent variables

*p < .05; **p < .01; ***p < .001

### Main Analyses

**Fig. 2:**
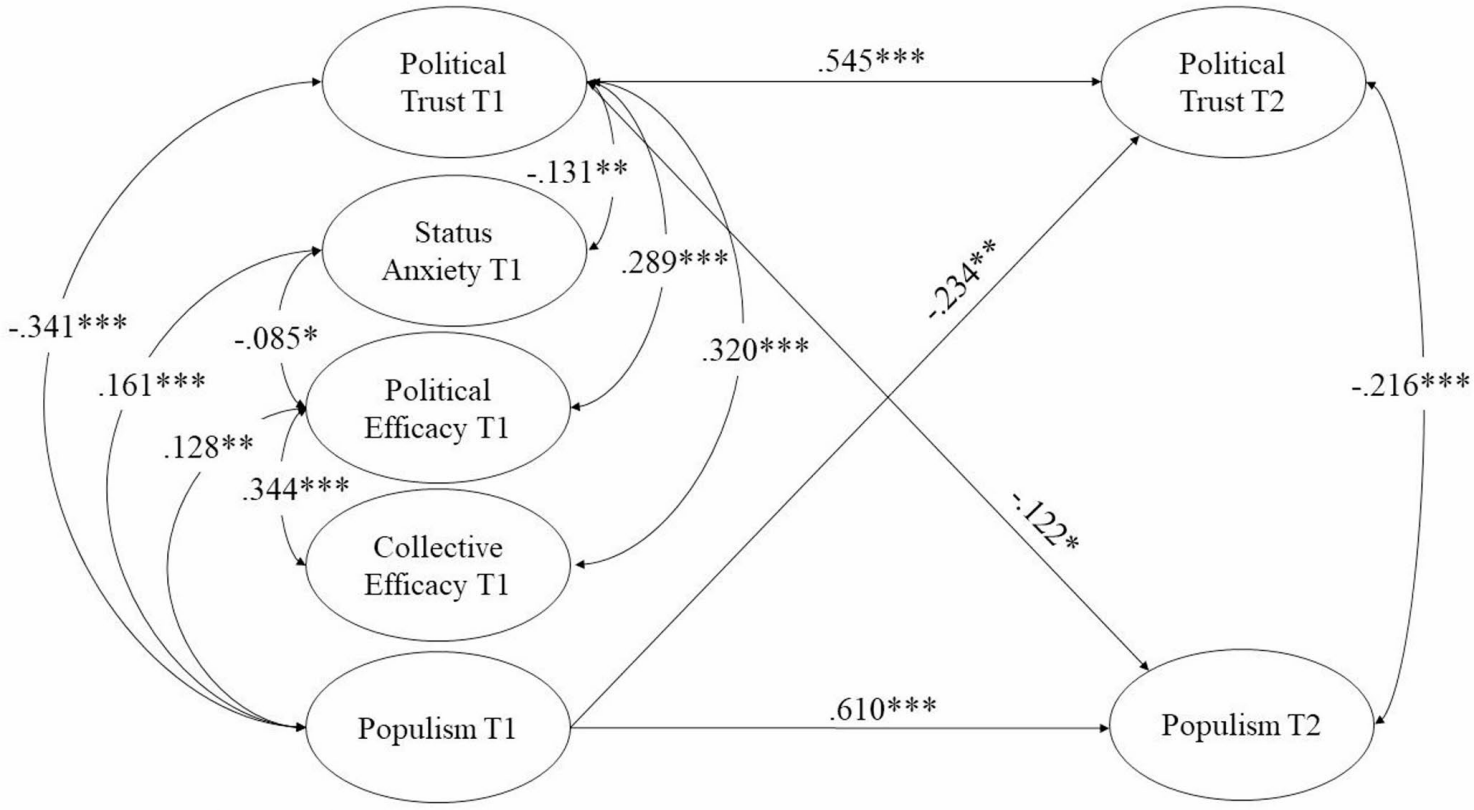
Structure equation model predicting political trust and populism over time. Note. Standardized coefficients. Not shown: Non-significant correlations and coefficients. All T1 variables wereused to predict political trust and populism at T2. Federal state, gender, and socioeconomic status are included aspredictors but not displayed to improve readability

Figure 2 shows the results of the latent structural equation model, which captures stability and cross-lagged paths between populist attitudes and political trust at T1 and T2, while relating both constructs at T2 to political efficacy, collective efficacy, and status anxiety at T1. In addition, students’ gender, socio-economic status, and the federal state were included as predictors with effects on all variables at T1 as well as political trust and populist attitudes at T2. Effects of these background variables and non-significant effects are not depicted in Fig. 2 to enhance readability (see Appendix B for a complete summary of the model). The model fit the data well (CFI=0.942; TLI = 0.929; SRMR=0.043; RMSEA=0.033) and explained a satisfying amount of variance in both political trust and populism (R²_Populism, T1_=0.053; R²_Trust, T1_=0.027; R²_Populism, T2_=0.441; R²_Trust, T2_=0.492). The results point to substantial rank-order stability in political trust and populist attitudes over time. These results indicate that both political trust and populist attitudes are already formed earlier in adolescence and are likely to persist.

As expected, populist attitudes and political trust were substantially negatively correlated at T1. Populism was also positively related to status anxiety and political efficacy, but not to collective efficacy. Additionally, political efficacy and collective efficacy were meaningfully linked. Status anxiety and political efficacy were only very weakly correlated.

The analyses further revealed significant cross-lagged effects linking trust at T1 with lower populist attitudes at T2, whereas populist attitudes at T1 predicted lower political trust at T2. The tested cross-lagged model controls for the variance of the variables introduced at T1. Therefore, significant paths on the T2 variables indicate changes in the residual variance, which can be interpreted as changes over time. Beyond this, neither political efficacy, collective efficacy, nor status anxiety were significantly related to populist attitudes or political trust at T2. Still, the relationships at T1 mainly reveal the expected pattern: Political trust is associated with lower status anxiety and higher internal as well as external efficacy. The anticipated association between populist attitudes and higher status anxiety at T1 was also confirmed. While no relationship was found for collective efficacy, a significant positive correlation emerged between political efficacy and populist attitudes. Individuals with higher political efficacy were more likely to endorse populist attitudes. Gender and socio-economic status had no predictive power for changes in political trust or populist attitudes between the two measurement points. The effects of these background variables on the T1 variables are very similar to the ones reported in Table 1.

### Sensitivity and Robustness Analyses

Given differences between the two federal states in terms of structural characteristics and prevailing political climates, a follow-up analysis was conducted to examine whether associations differed across states. To this end, multiple-group modeling was employed, in which the effects of collective efficacy, internal political efficacy, and status anxiety at T1 on political trust and populist attitudes at T2 were freely estimated, while controlling for the stability of both outcomes from T1 (i.e., unconstrained model). Subsequently, all effects were constrained to be equal across both federal states (i.e., constrained model). Model comparisons indicated that the more parsimonious constrained model did not fit the data significantly worse than the unconstrained model, which was therefore retained; Δχ²(17) = 23.81, *p* = .125, ΔCFI = 0.001, ΔRMSEA < 0.001. Accordingly, there was no indication that the associations were moderated by federal state.

Germany has a tracked school system, in which students attend different types of schools that prepare them either for university or vocational training (academic vs. practically oriented schools). To account for this, sensitivity analyses including school type as additional covariate were conducted, differentiating between students attending the academic track and those who did not. While controlling for school type did not change the main results, the following covariate effects were observed: students in practically oriented schools reported lower political trust T1 (β = − 0.146; *p* = .001) but school type did not relate to populism at T1 or to changes in trust or populism between T1 and T2. Students at practically oriented schools additionally reported higher levels of status anxiety (β = 0.083; *p* = .023) and lower levels of political efficacy (β = − 0.126; *p* = .005). School type was not included in the main analysis, as it is strongly related to students’ socio-economic-status (Schindler, 2017), which was controlled for in the analysis.

Similarly, the immigration history of students’ families is confounded with the socio-economic status, the school type, and federal state (Autor: innengruppe Bildungsberichtserstattung, 2024). Students that migrated or have one parent that migrated were less likely to live in Thuringia or to attend the academic tracks. When the immigration background was introduced as additional predictor, it negatively predicted trust in political institutions (β = − 0.114; *p* = .004) and stronger status anxiety (β = − 0.108; *p* = .007). No differences in populism at T1 or changes in populism or trust related to the immigration history.

## Discussion

Populist movements and parties have been building their foundations for decades and are likely to last (Mudde, 2013). Populist modes of thinking conflict with educational efforts aimed at enabling students to analyze societal challenges from multiple perspectives (Abs, 2021). To inform policy makers and practitioners, researchers need to recognize the formation and development of populist attitudes in the contexts of education. The present study locates populist attitudes within the political mindset by conceptually and empirically separating them from political trust. While the revocation of political trust can foster democratic reforms (Norris, 2011), populist attitudes are connected to a strong devaluation of any political opposition. This is challenging for pluralistic societies that treat the inclusion even of contrary views as potentially beneficial for problem-solving processes.

### Longitudinal Effects Between Populist Attitudes and Political Trust

Using longitudinal data from 1,206 Grade 9 students, we were able to show that populist attitudes can be measured well across two measurement points and that they show a substantial level of stability among German teenagers. The stability and changes across a school year were assessed. We found populist attitudes and political trust to be relatively, but not overwhelmingly stable. While youth is a time of changes in cognitive capabilities and attitudes, political trust and populist attitudes were somewhat stable. Greater changes might be found in analyses that measure changes across multiple years.

The analyses further showed that populist attitudes are related to, yet distinct from (a lack of) political trust (H1a); and that populist attitudes and political trust mutually affected each other across a school year (H1b). The undermining force of populist attitudes towards political trust is not unexpected but still noteworthy, as change in political trust was not predicted by any other variable analyzed here. Previous research implies that trust can be fostered through other means (e.g., by improving the school climate; Ziemes, 2025). Political efficacy, collective efficacy, and future anxiety were used to predict political trust and populist attitudes at T2 but despite their clear cross-sectional relationships, the variables did not explain change in either dependent variable.

### The Position of Populism Within the Political Mindset

This study positions populist attitudes within the broader political mindset of adolescents and links them to political beliefs that are considered relevant for the formation of populist attitudes. As such, a sense of political help- or hopelessness is often thought to turn people towards populist narratives (Sant, 2021). While populist attitudes were significantly correlated with status anxieties and political efficacy (as expected in H2a), status-related anxieties did not predict changes in populist attitudes or political trust over time. Neither did internal efficacy or collective efficacy affect populist attitudes or political trust (H2b). The presented results, thus, do not support earlier research showing that status anxieties fuel populist attitudes but rather indicate that populist attitudes and future anxieties coincide among emerging citizens. As political trust negatively predicted populist attitudes and correlated negatively with future anxieties, it is reasonable to assume that a lack of political trust can lead to an increase in status anxiety as well. As described above, political trust contains the belief that the institutions in power will not act against one’s interests (Norris, 2022). If these institutions are perceived to work against oneself – or in favor of societal groups seen as a threat – status anxiety may arise, accompanied by a populist way of thinking.

Both internal and collective efficacy revealed expected associations with higher levels of political trust. The pattern of associations was more nuanced for students’ populist attitudes: No significant links emerged with collective efficacy, yet youth who endorsed populist beliefs felt more politically competent and efficacious. This latter association may be explained by the connotation of the construct: A key component of populism involves confident attributions of one’s own political competence (Mudde, 2004). Surprisingly, these associations were reflected in individual rather than collective efficacy beliefs, which may be because the latter specifically referred to the impact of young people as a group rather than to “the people” in a broader sense. For future research, it is important to examine these patterns more thoroughly and to explore potential dynamics between collective and individual political efficacy beliefs in greater depth.

Finally, among the considered socio-demographic covariates, one effect warrants mention: The region or federal state remained a significant predictor of populism throughout the study. Students in Thuringia, a region located in the former GDR, held stronger populist attitudes compared to their peers, even after controlling for their gender^5^ and socio-economic status. Many inhabitants of the former GDR perceive themselves as second-class citizens and socially disadvantaged (Hartleb, 2022). While it is beyond this study to determine whether differences stem from historical developments, current opportunity structures, or variations in political culture, the results support the notion that populist attitudes are also shaped by macro-contextual circumstances and may be sensitive to feelings of devaluation. Further research comparing different regional contexts is necessary to better understand the relevance of demographic characteristics for the formation of populist attitudes in youth.

### Study Limitations

The present study contributes to the literature by examining populist attitudes in youth and their position within the political mindset across time. Still, several limitations need to be noted: As only two measurement points were available and no experimental manipulation was conducted, we can only make limited assumptions concerning the causal directions of the relationships considered. While multiple relevant variables concerning the political mindset were included, previous studies indicate that further variables – such as anger, misinformation, or social media consumption – might also play important roles (Soares et al., 2024). The generalizability of our research is increased by drawing on data from two different federal states of Germany with varying historical and cultural contexts. Still, the data is mainly based on a convenience sample and is, therefore, not representative of either region in general. Additionally, the attrition was not random. Students with a lower socio-economic status and less trust were less likely to participate in both points of measurement. If students that distrust the state are more likely to drop out, this could pose a challenge for research on political socialization in general. The analyses indicate that political trust and populism are profoundly linked. If students declined to participate at T2 out of distrust towards the researchers or the political system in general, it is possible that the link between political trust and populism is under- rather than overestimated here. Previous research has shown that the trustworthiness of a context can influence the formation of political trust (Stals & Ziemes, 2024). The strength of regional institutions and opportunity structures might also interact with the formation of political attitudes.

Moreover, some limitations related to the measurement instruments should be mentioned. Full metric measurement invariance was established for both outcome variables, but scalar invariance could not be confirmed for political trust and was only partially achieved for populism. While this may affect the comparability of latent means across time, with our focus on relative changes, this limitation is unlikely to substantially affect the interpretation of the results. Finally, it should be noted that the current study captured populism through a (latent) composite mean score, based on a shortened measurement instrument originally developed for adult samples (Schulz et al., 2018). The confirmatory factory analyses conducted as well as the zero-order correlations support the assumption that the construct was measured successfully. A dedicated validation study of the used scale would still be very desirable. From a theoretical perspective, the construct’s multidimensional nature should be taken into account. As such, populism is assumed to reflect a latent construct emerging from the interplay of the subdimensions of anti-elitism, anti-pluralism, and popular sovereignty (Mudde, 2004). Research suggests that populism can be reliably measured as a multidimensional construct in late adolescence with the facet of anti-elitism at its core (Grunwald et al., under review). While our measure also focused on the dimension of anti-elitism, future studies should investigate whether populism’s multidimensional nature can be reliably confirmed within younger age groups, as in this study.

Finally, socialization agents such as the parents, peers, and the school were not considered, although they could play an important role for the formation of populist attitudes. While this study focused on the position of populist attitudes within the political mindset and internal predictors of change over time, future research should account for the relevance of key socialization agents in more detail (Jungkunz & Weiss, 2024; Noack & Eckstein, 2023). This study included students’ perception of their families’ socio-economic status as a control variable, but not parental attitudes or experiences with peers.

### Study Implications

One key starting point for prevention is the school context: Practitioners can foster a positive social climate that strengthens connections between different social groups. Furthermore, young people can be encouraged to openly address controversial political and social issues, engage with diverse perspectives, and form their own opinions (Noack & Eckstein, 2023). Accordingly, expected links were reported between the experience of a democratic school setting and lower political cynicism as well as higher political trust (Claes et al., 2012; Tzankova et al., 2021), which may also contribute to countering populism. In this regard, associations between populist attitudes and the quality of teacher-student relationships have already been shown (Jungkunz & Weiss, 2024). However, further – especially longitudinal – studies are needed to provide a more comprehensive understanding of the underlying dynamics.

The presented results point to important implications for practice and policy: As populist attitudes can undermine political trust, teachers should be sensitive to potential populist statements of students and foster a thorough understanding of core democratic ideas. Sant (2021) stresses the importance of teaching students to acknowledge different perspectives, articulate their own perspectives, and to participate within their immediate (e.g., school) as well as broader social environments (i.e., society). These pedagogical approaches have yet to be evaluated in greater depth concerning their potential effects on populist attitudes. However, given that a core feature of populist attitudes is the construction of issues as dichotomous or even Manichean rather than multipolar (Schäfer, 2022), any educational approach that presents *more than two distinct perspectives* on social and political issues could be particularly relevant. Furthermore, differentiating between populism and distrust teachers may foster critical citizenship, which enables students to investigate the merit of the institutions and incumbents they judge. From a policy perspective, these findings are relevant as they demonstrate that populist attitudes are an important topic in adolescence and that unattended populistic attitudes in youth might undermine political trust even before emerging citizens gain the right to vote in elections.

## Conclusion

There is little research concerning the origins and early development of populist attitudes in youth and how they can be distinguished from political trust. Present results show that both attitudes can be clearly distinguished and are negatively connected over time, while other political attitudes, i.e. status anxiety, political efficacy, and collective efficacy only show cross-sectional relations While political trust predicts a reduction in populist attitudes, an isolated fostering of political trust to reduce early populist attitudes needs to be considered with great caution. Strengthening political trust in emerging citizens without providing them with the tools to judge institutions and incumbents on their merits should be seen as a form of propaganda (Kloubert, 2018) and therefore be avoided. The present results suggest that populist attitudes in emerging citizens hinder the development of critical citizenship, i.e. the ability to evaluate institutions using their acquired understanding of democratic principles and their values. Instead, their populist attitudes decrease political trust. Further research is needed to identify effective means of preventing and reducing populist attitudes while enabling students to engage critically with political processes and institutions. Overall, the analyses show that within the political mindset of 14-year-old students, populist attitudes are already a consolidated set of political views. The stability underlines that the original formation of populist attitudes starts early in life, during developmental periods in which students possess fewer cognitive prerequisites to reflect and critically evaluate civic processes, instructions, and their actors, and are less able to judge institutions and incumbents based on their merits.

## Supplementary Information

Below is the link to the electronic supplementary material.

Supplementary Material 1

## Data Availability

The datasets generated and/or analyzed during the current study are not publicly available but can be obtained from the second author upon reasonable request.

## Footnotes

1 If populists are in power, they will shift their distrust to institutions they are not (yet) dominating, perpetuating a climate of distrust and defensiveness despite their dominance (see Pirro & Taggart, 2023).

2 Only weak connections between political trust and internal political efficacy have been found so far and the strength of the connections did not clearly relate to political context factors investigated (see Torney-Purta et al., 2004).

3 Intraclass correlations at the classroom level were as follows: Populist attitudes: ICC_T1 = 0.057, ICC_T2 = 0.036; political trust: ICC_T1 = 0.024, ICC_T2 = 0.054; political efficacy: ICC = 0.037; collective efficacy: ICC = 0.045; status anxiety: ICC = 0.032.

4 Partial scalar invariance across time could be established after freely estimating the intercepts of the items ‘People like me have no influence on what the government does’ and ‘Politicians should listen more to the public’.

5 In contrast to earlier studies, we did not find a substantial connection of populist attitudes and gender.
