# Supplementary material for: Populist Attitudes in Adolescence are Measurable, Stable, and Linked to Political Trust: A Longitudinal Analysis of German High-School Students
Source: J Youth Adolesc. 2026 Mar 6;55(6):1418–32. doi: 10.1007/s10964-026-02332-x (PMC13263212; doi:10.1007/s10964-026-02332-x)
Supplement: Supplementary file 1 — Supplementary Material 1 [file 10964_2026_2332_MOESM1_ESM.docx]

**Appendix A**

**Item wordings**

**Table A1**

Items of the populist attitude scale

|  | T1 | | T2 | |
| --- | --- | --- | --- | --- |
|  | *M* | *SD* | *M* | *SD* |
| The members of parliament lose touch with the people quite quickly. | 3.41 | 0.93 | 3.45 | 1.00 |
| The differences between the people and the so-called elite are much bigger than the differences within the group of the people. | 3.23 | 0.90 | 3.33 | 0.90 |
| People like me have no influence on what the government does. | 3.34 | 1.25 | 3.56 | 1.20 |
| Politicians should listen more to the public. | 3.93 | 0.84 | 3.85 | 0.88 |
| A few powerful people decide the fate of millions of people. | 3.57 | 1.16 | 3.61 | 1.11 |

1= *disagree completely*; 5= *agree completely*.

**Table A2**

Items of the political trust scale

|  | T1 | | T2 | |
| --- | --- | --- | --- | --- |
|  | *M* | *SD* | *M* | *SD* |
| … the government | 2.74 | 0.93 | 2.65 | 0.94 |
| … political parties | 2.78 | 0.86 | 2.59 | 0.88 |
| … the European Union (EU) | 3.21 | 0.90 | 3.13 | 0.95 |

Stem: How much do you trust…1= *not at all*; 5= *fully*.

**Table A3**

Items of the status anxiety scale

|  | *M* T1 | *SD* T1 |
| --- | --- | --- |
| Unemployment or worsening of my parents’ financial situation. | 3.65 | 1.23 |
| Not finding an apprenticeship or employment. | 3.72 | 1.26 |
| Being financially worse off than my parents | 3.35 | 1.34 |

Stem: Different people regard some things as a big problems that others believe is a minor matter. When you think about your future, how much do the following things worry you? 1= *worries me not at all*; 5=*worries me a lot*.

**Table A4**

Items of the political efficacy scale

|  | *M* T1 | *SD* T1 |
| --- | --- | --- |
| It is easy for me to take part in discussions about political issues. | 3.10 | 1.19 |
| I feel able to engage in political issues. | 2.54 | 1.19 |
| I believe I have a fairly good understanding of important political issues. | 3.04 | 1.06 |

1= *disagree completely*; 5= *agree completely*.

**Table A5**

Items of the collective efficacy scale

|  | *M* T1 | *SD* T1 |
| --- | --- | --- |
| I believe young people can change things if they work together. | 4.11 | 0.92 |
| If young people cooperate, they can influence the decisions of the government. | 3.39 | 1.09 |

1= *disagree completely*; 5= *agree completely*.

**Appendix B**

**Multivariate robustness analysis**

**Table B1**

Results of the latent structure equation model

| **Populism at T2 regressed on** | β / *r* | *SE* |
| --- | --- | --- |
| Populism T1 | .610*** | .057 |
| Political trust T1 | -.122* | .055 |
| Status anxiety T1 | .014 | .036 |
| Political efficacy T1 | -.018 | .056 |
| Collective efficacy T1 | -.003 | .045 |
| Federal state (TH=1) | .001 | .039 |
| Gender (M=1) | -.007 | .034 |
| Socioeconomic status | .032 | .024 |
|  |  |  |
| **Political trust at T2 regressed on** |  |  |
| Populism T1 | -.234*** | .053 |
| Political trust T1 | .545*** | .058 |
| Status anxietyT1 | -.019 | .043 |
| Political efficacy T1 | .029 | .027 |
| Collective efficacy T1 | .079 | .045 |
| Federal state (TH=1) | .023 | .035 |
| Gender (M=1) | .028 | .033 |
| Socioeconomic Status | .008 | .033 |
|  |  |  |
| **Populism at T1 regressed on** |  |  |
| Federal state (TH=1) | .201*** | .055 |
| Gender (M=1) | -.061 | .041 |
| Socioeconomic Status | -.087* | .037 |
|  |  |  |
| **Political trust at T1 regressed on** |  |  |
| Federal state (TH=1) | -.084* | .040 |
| Gender (M=1) | .090* | .035 |
| Socioeconomic Status | .106*** | .030 |
|  |  |  |
| **Correlations** |  |  |
| Populism T1 / Political trust T1 | -.341*** | .051 |
| Populism T2 / Political trust T2 | -.219*** | .051 |
| Status anxiety / Populism T1 | .161*** | .037 |
| Status anxiety / Political trust T1 | -.131** | .043 |
| Political efficacy / Populism T1 | .159** | .048 |
| Political efficacy / Political trust T1 | .289*** | .040 |
| Political efficacy /Status anxiety | -.085* | .038 |
| Collective efficacy / Populism T1 | .082 | .059 |
| Collective efficacy / Political trust T1 | .329*** | .046 |
| Collective efficacy / Status anxiety | .033 | .043 |
| Collective efficacy / Political efficacy | .344*** | .051 |
| Federal state/ Status anxiety | .098* | .048 |
| Federal state / Political efficacy | -.083 | .049 |
| Federal state / Collective efficacy | -.144** | .054 |
| Gender / Status anxiety | -.201*** | .034 |
| Gender / Political efficacy | .028 | .036 |
| Gender / Collective efficacy | -.157** | .047 |
| Gender / Federal state | -.027 | .043 |
| Socioeconomic status / Status anxiety | -.182*** | .042 |
| Socioeconomic status / Political efficacy | .066 | .034 |
| Socioeconomic status / Collective efficacy | .069 | .044 |
| Socioeconomic status / Federal state | -.045 | .044 |
| Socioeconomic status / Gender | -.044 | .047 |
|  |  |  |
| R² Political trust T1 | .027 | .012 |
| R² Populist attitudes T1 | .053 | .023 |
| R² Political trust T2 | .492 | .048 |
| R² Populist attitudes T2 | .441 | .060 |
|  |  |  |
| CFI | .942 |  |
| TLI | .929 |  |
| SRMR | .043 |  |
| RMSEA | .033 |  |
| Chi² of model fit | 660.405 |  |
| df | 283 |  |
|  |  |  |
| *N* | 1206 |  |
| *Note.* **p*<.05; ***p*<.01;****p*<.001; M=Male; NRW = North Rhine-Westphalia; TH = Thuringia. | | |
